# Mobbing, Gender Roles, and Job Satisfaction Among Healthcare Radiation Workers: A Cross-Cultural Study

**DOI:** 10.3390/healthcare14121610

**Published:** 2026-06-08

**Authors:** Meryem Cansu Şahin, Ebru Başkaya, Mihaela Tinca Udriștioiu

**Affiliations:** 1 Department of Medical Services and Techniques, Vocational School of Health Services, Usak University, 1 Eylül Campus, 64200 Uşak, Türkiye; meryem.sahin@usak.edu.tr; 2 Department of Psychiatric Nursing, Faculty of Health Sciences, Usak University, 1 Eylül Campus, 64200 Uşak, Türkiye; ebru.kurdal@usak.edu.tr; 3 Department of Physics, Faculty of Science, University of Craiova, 13 A.I. Cuza Street, 200585 Craiova, Romania

**Keywords:** job satisfaction, workplace mobbing, healthcare radiation workers, social gender perception, cross-cultural comparison

## Abstract

Background/Objectives: Workplace mobbing represents a psychosocial occupational hazard for healthcare radiation workers and highlights the need to consider gender and cultural factors in radiology departments. This study examines the links between mobbing, gender role attitudes, and job satisfaction among healthcare radiation workers and compares these across Türkiye and Romania. Methods: In this cross-sectional, culturally comparative study, 191 healthcare radiation workers (Türkiye: *n* = 104; Romania: *n* = 87) completed surveys assessing the Gender Roles Attitude Scale, Mobbing Scale, and Job Satisfaction Scale. Group comparisons were conducted using independent-samples tests, and correlations were assessed using Pearson’s correlation coefficient. Hierarchical multiple linear regression was used to identify predictors of job satisfaction. Results: In Türkiye, job satisfaction was negatively associated with mobbing scores (r = −0.551, *p* < 0.001). In Romania, job satisfaction was positively associated with gender role attitudes (r = 0.346, *p* < 0.001) and negatively associated with mobbing scores (r = −0.501, *p* < 0.001). In addition, gender role attitudes were negatively associated with mobbing scores (r = −0.398, *p* < 0.001). Conclusions: These findings demonstrate that workplace mobbing is associated with job satisfaction in both countries, while gender role attitudes showed different correlation patterns across the two country samples. Only in Romania were attitudes towards gender roles associated with job satisfaction. Context-specific strategies targeting workplace mobbing, gender perceptions, and work organization to enhance psychosocial well-being and workforce stability in radiology departments.

## 1. Introduction

Medical imaging, radiotherapy, and nuclear medicine departments are complex, high-risk environments in which healthcare radiation workers play a critical role in ensuring diagnostic accuracy and patient safety. These settings require sustained attention, technical precision, and strict adherence to radiation protection principles. In addition to physical hazards, healthcare radiation workers might be exposed to significant psychosocial stressors that might impair their concentration, decision-making, and compliance with safety protocols, thereby increasing the risk of occupational exposure and compromising patient safety. Healthcare radiation workers hold certifications to work in this field and have distinct legal rights [1,2].

Gender inequality refers to discrimination based on gender that restricts individuals from reaching their potential, pursuing careers, or making free choices, thereby impeding gender equality. Achieving gender equality, the UN Sustainable Development Goal 5, is important for promoting women’s rights, economic empowerment, poverty reduction, and fostering a sustainable, peaceful world [3,4,5].

Mobbing is systematic psychological harassment against employees, and it manifests itself through behaviours such as humiliating, excluding, and constantly criticizing the person [6]. Professionals working with ionizing radiation may be more vulnerable to mobbing and gender-based inequality due to the inherently high-risk and high-responsibility nature of their work. The need for strict radiation protection protocols, combined with long and irregular working hours, can contribute to increased occupational stress and hierarchical pressure in clinical environments. These conditions may exacerbate interpersonal conflicts and power imbalances, particularly in healthcare settings where gender disparities in leadership positions persist [7]. Mobbing triggers the development of psychological disorders, such as stress and anxiety, among radiation workers, resulting in decreased job satisfaction and burnout syndrome [8].

Job satisfaction refers to the degree of satisfaction or dissatisfaction people feel about their jobs. According to the Job Demand–Resources (JD-R) model, adverse working conditions, such as excessive workload, interpersonal conflict, and psychological harassment, function as job demands that deplete employees’ psychological resources, ultimately reducing job satisfaction and increasing burnout [9].

Radiation-related departments are traditionally male-dominated, and female employees may be exposed to more discrimination and gender-based injustice in such workplaces [10]. Female employees are at greater risk of mobbing than their male counterparts because gender roles and expectations place women at a more significant disadvantage in the workplace [11]. Studies conducted among radiation workers show that women are more isolated, excluded, and subjected to more invisible forms of mobbing at work [12]. This situation adversely affects both women’s professional and personal development.

The effects of gender-based discrimination and mobbing have adverse effects not only on the mental health of individuals but also on the overall performance and culture of the workplace [13,14]. Because radiation workers work in challenging and hazardous conditions, a strong sense of trust and workplace support is essential. However, mobbing and gender inequality erode trust, undermining employees’ motivation and commitment [7,15]. Research has shown that the long-term consequences of mobbing include adverse outcomes such as anxiety, depression, burnout, and intention to leave work [16,17,18].

Despite increasing research on workplace mobbing and gender inequality in healthcare, workers in ionizing radiation environments are often overlooked. Medical imaging, radiotherapy, and nuclear medicine departments are marked by high workloads, shift schedules, significant occupational hazards, and male-dominated cultures, which heighten psychosocial stress for workers. So far, little empirical research has explored how workplace mobbing, gender perceptions, and job satisfaction intersect among radiation workers.

This study explores the connections between workplace mobbing, gender role attitudes, and job satisfaction among healthcare radiation workers. It compares these relationships in Türkiye (a non-EU country) and Romania (an EU member state). Adopting a cross-cultural approach that considers psychosocial and socio-cultural factors, the research aims to deepen understanding of workplace well-being and job satisfaction in radiation healthcare environments.

## 2. Materials and Methods

This study examined the relationships among gender role attitudes, mobbing experiences, and job satisfaction among radiation workers in Türkiye and Romania and descriptively compared these associations across the two country samples.

### 2.1. Study Design and Research Hypothesis

This cross-sectional comparative study aimed to examine the relationships among workplace mobbing, gender role attitudes, and job satisfaction among healthcare radiation workers in Türkiye and Romania. Consistent with the aims of the study and the statistical analyses performed, the following hypotheses were formulated:

**H1.**  *Workplace mobbing is negatively associated with job satisfaction among healthcare radiation workers.*

**H2.**  *More egalitarian gender role attitudes are positively associated with job satisfaction among healthcare radiation workers.*

**H3.**  *Workplace mobbing is negatively associated with egalitarian gender role attitudes among healthcare radiation workers.*

**H4.**  *Descriptive differences in the associations among workplace mobbing, gender role attitudes, and job satisfaction may be observed between the Türkiye and Romania samples.*

### 2.2. Population and Sample

This sample comprises 191 healthcare radiation workers from Türkiye and Romania, across various healthcare institutions in both countries (104 from Türkiye and 87 from Romania).

An a priori sample size analysis was conducted using G*Power 3.1.9.7 [19]. Assuming a medium effect size (r = 0.35) based on Cohen’s recommendations for behavioral sciences research [20] a significance level of α = 0.05, and a statistical power of 95% for the primary analyses examining associations between study variables, the minimum required total sample size was calculated as 79 participants. To increase statistical power and support cross-country comparisons, a total of 191 healthcare radiation workers were ultimately included in the study. The inclusion criterion required participants to have worked in their institution for at least 6 months, as this duration was considered sufficient to evaluate potential exposure to mobbing behaviors [21].

The professional composition of the samples naturally reflected the workforce profiles of the participating institutions, with radiographers more frequently represented in Türkiye and medical physicists more frequently represented in Romania. In Türkiye, radiographers primarily perform imaging and radiotherapy-related procedures that involve direct patient interaction, including patient positioning and operating imaging equipment. In contrast, medical physicists in Romania are mainly responsible for radiation safety, treatment planning, dose calculations, quality assurance, and technical supervision of radiation-based procedures. Medical physicists do not have direct patient interaction. Nurses are involved in imaging and radiotherapy procedures, including the injection of contrast agents and patient positioning. Technicians are responsible for operating the imaging equipment and the linear accelerator. Although these professions differ in their specific responsibilities and educational backgrounds, both work in ionizing radiation environments and face similar organizational and occupational stressors. This diversity provides a broader perspective on psychosocial experiences within medical imaging, radiotherapy, and nuclear medicine departments and should be considered when interpreting cross-country comparisons. Although nurses and medical doctors were not the predominant professional groups in the study, they were retained in the analyses because they represented a very small proportion of the total sample. Excluding these participants would have further reduced the already limited sample size and statistical power. To minimize potential occupational confounding, profession was included as a control variable in the regression analyses.

### 2.3. Survey, Data Collection Process and Tools

A 14-question survey was developed by researchers in accordance with the literature. It included participants’ age, gender, marital status, whether they had children, profession, employment interruption, years of work, gender-based disadvantages when being hired, use of legal leave on the requested date, gender-related disadvantages when determining the leave dates, marital status-related disadvantages when determining the leave dates, gender-related disadvantages when planning working hours, marital status-related disadvantages when planning working hours, and information about the institution they worked.

The data were collected in this study between 30 July and 15 October 2024. Data collection was conducted simultaneously in both countries. Participation in the study was voluntary. A Google Form was distributed via closed professional social media groups affiliated with professional associations. A convenience sampling strategy was used due to the difficulty of reaching radiation workers across multiple institutions and the relatively small size of this professional population. This approach is commonly preferred in occupational health studies where the target group is limited and geographically dispersed.

The same online recruitment strategy was used in both Türkiye and Romania. The questionnaire link was distributed through professional association communication channels and closed professional social media groups involving healthcare radiation workers. Participation was restricted to individuals working in medical imaging, radiotherapy, or nuclear medicine departments. Eligibility was assessed using self-reported professional and workplace information collected in the demographic section of the survey.

Google Forms settings were configured to limit multiple submissions from the same account, and incomplete questionnaires were excluded prior to analysis. Because the survey was distributed through multiple online professional networks and closed groups, the exact number of individuals who received the survey invitation could not be determined. Therefore, a response rate could not be calculated.

The tools Gender Roles Attitude Scale (GRAS), Job Satisfaction Scale (JSS), and Mobbing Scale (MS) were used in this study to collect data.

#### 2.3.1. Gender Roles Attitude Scale (GRAS)

The Gender Role Attitude Scale (GRAS), developed by Zeyneloğlu in 2008, was designed to assess university students’ attitudes toward gender roles [22]. The author concluded that there are no purely traditional attitudes among women or men about gender roles, indicating a gradual transition towards egalitarian views in Romanian society. The scale is a five-point Likert-type scale. GRAS comprises 38 items and five sub-dimensions. The internal consistency of the scale was 0.92 (Cronbach’s alpha). Related to GRAS, Cronbach’s alpha was 0.89 in the Turkish sample and 0.91 in the Romanian sample.

#### 2.3.2. Job Satisfaction Scale (JSS)

The adaptation of the short form of the Job Satisfaction Scale (JSS), developed by Brayfield and Rothe (1951), and the 5-item short form created by Judge, Locke, Durham and Kluger (1998), into Turkish, along with an examination of its psychometric properties and a validity and reliability study, were conducted by Keser and Bilir (2019) [23,24,25]. In the validity and reliability study, factor analysis indicated that a single-factor structure of the JSS was appropriate for the Turkish employee sample. The internal consistency reliability of the scale was 0.85 (Cronbach’s alpha). As a result, the psychometric properties of the JSS were evaluated. JSS was found to be a valid and reliable tool for measuring employees’ job satisfaction. Stoica-Constantin et al. adapted the Job Satisfaction Scale to Romania, examined its psychometric properties, and conducted a validity and reliability study [26]. The JSS was chosen for its extensive use in organizational psychology and healthcare workforce studies, its robust psychometric properties, and its sensitivity to detecting variations in job satisfaction in demanding work environments. Its short form further facilitates reliable measurement in large-scale field studies, such as the present research. For JSS, the Cronbach’s alpha values were 0.90 in the Turkish sample and 0.76 in the Romanian sample.

#### 2.3.3. Mobbing Scale (MS)

The Mobbing Scale (MS), originally developed by Aiello et al., was used in both Türkiye and Romania to ensure cross-cultural comparability. For the Turkish sample, the validated Turkish adaptation developed by Özmete and Laleoğlu was used [27,28]. The internal consistency reliability of the scale was 0.95 (Cronbach’s alpha). For the Romanian sample, the same instrument was translated into Romanian using a forward–backward translation procedure conducted by bilingual experts. Semantic and conceptual equivalence between the Turkish and Romanian versions was reviewed before data collection.

The scale consisted of the same 38 items and five subdimensions in both countries: relationships with co-workers, threats and harassment, obstacles related to work and career, interference with private life, and commitment to work. All items were scored using the same 7-point Likert response format, and higher scores indicated greater exposure to mobbing. Therefore, total scores were directly comparable across countries. Related to MS, Cronbach’s Alpha was 0.95 in the Turkish sample and 0.96 in the Romanian sample.

### 2.4. Ethical Considerations

This study was conducted in accordance with the Declaration of Helsinki and complied with confidentiality, data collection, and data processing regulations in both countries. Ethical approval for this study was obtained from the Social and Human Sciences Scientific Research and Publication Ethics Committee at Uşak University prior to survey administration. This approval covered the international collaborative research process, including data collection involving participants from Romania, conducted in collaboration with the University of Craiova. Before data collection, participants were informed of the purpose and scope of this study and provided informed consent. The “Informed Consent Form” was added to Google Forms, and participants had to select the voluntary consent option to continue the survey. The average time to complete the survey was 15 min.

### 2.5. Data Analysis

For data analysis, the authors used SPSS (Statistical Package for the Social Sciences), version 22 (IBM Corp., Armonk, NY, USA) [29]. Descriptive statistical methods (mean, standard deviation, and percentage distribution) were used to analyze the data.

For quantitative data, one-way ANOVA and the two-independent-groups *t*-test were used to compare normally distributed parameters between groups. The Kruskal-Wallis test was used to compare parameters that did not follow a normal distribution across groups, and the Mann-Whitney U test was used for two-group comparisons. Pearson correlation analysis was used to evaluate the relationships between variables. Skewness and kurtosis values of ±2 were used to assess normality [30]. Pearson correlation analysis was preferred because skewness and kurtosis values were within acceptable ranges (±2), indicating approximate normality of the continuous variables. The results were evaluated using a 95% confidence interval and a significance level of *p* < 0.05. Effect sizes were calculated to assess the magnitude of group differences. Cohen’s d values were reported for independent-samples *t*-tests, η^2^ values for one-way ANOVA analyses, and r effect sizes for Mann–Whitney U tests. Because the analyses presented in results involved multiple exploratory comparisons, the results should be interpreted cautiously due to the potential risk of Type I error.

In addition, a hierarchical multiple linear regression analysis was used to identify predictors of job satisfaction. Variables were introduced into the regression models in two steps: mobbing, gender role attitudes, and country were included in Model 1, while age, gender, years of professional experience, and profession were added in Model 2 to assess their additional contribution. Residual normality, homoscedasticity, and independence of errors were evaluated prior to interpreting the regression models. All VIF values were below 10, and tolerance values were above 0.10, indicating no evidence of problematic multicollinearity. Independence of errors was supported by a Durbin–Watson value of 1.97. Standardized residual ranged from −3.18 to 2.87, suggesting no severe violations of residual normality or homoscedasticity assumptions.

## 3. Results

Table 1 summarizes the socio-demographic and occupational characteristics of the participants. The sample in both countries was predominantly female, with a higher proportion in Romania. Marital status differed between groups, with more single participants in Türkiye and more married participants in Romania. Most participants had no children and had experienced a work break in both countries.

Occupational distribution varied markedly, with technicians predominating in Türkiye and medical physicists in Romania. A substantial proportion of participants in both countries had more than 11 years of professional experience.

In terms of workplace perceptions, most participants reported no gender-based disadvantage during recruitment or in work organization processes, with consistently higher rates in Romania. However, difficulties in using legal leave were more frequently reported in Türkiye.

Table 2 presents the overall and subdimension scores of the GRAS, MS, and JSS among radiation workers. In the Turkish sample, the highest mean GRAS subdimension scores were observed in egalitarian and marriage-related gender roles, whereas the lowest mean scores were observed in traditional and male gender roles. Within the mobbing scale, the highest mean MS subdimension score was observed for relationships with co-workers, whereas the lowest mean scores were observed for commitment to work and interference with private life. The mean JSS score was lower in the Turkish sample than in the Romanian sample.

In the Romanian sample, the highest mean GRAS subdimension scores were observed in egalitarian and marriage-related gender roles, whereas the lowest mean scores were observed in traditional and male gender roles. Regarding mobbing, relationships with co-workers had the highest mean score among the MS subdimensions. Higher mean scores were observed for interference with private life and commitment to work compared with the Turkish sample, whereas lower mean scores were observed for work- and career-related obstacles. The mean JSS score was higher in the Romanian sample than in the Turkish sample.

Table 3(a) presents the comparison of JSS, GRAS, and MS scores by demographic characteristics. Among Turkish participants, statistically significant differences in GRAS mean scores were observed according to gender. In the Romanian sample, GRAS mean scores varied significantly according to gender and occupation. No significant differences were found in JSS or MS scores based on demographic characteristics in either sample, with the exception of years of service among Turkish participants, which was associated with significant differences in JSS mean scores.

Table 3(b) presents the comparison of JSS, GRAS, and MS scores by workplace conditions and gender-related experiences. Among Turkish participants, significant differences in JSS mean scores were observed in relation to the ability to use legal leave at a preferred time, marital status disadvantages in determining leave dates, gender-related disadvantages in planning working hours, and marital status disadvantages in planning working hours. GRAS mean scores differed significantly according to gender-related disadvantages during hiring and in determining leave dates. MS mean scores showed significant differences associated with taking a break from work, gender-related disadvantages during hiring, the ability to use legal leave at a preferred time, and disadvantages related to both gender and marital status in determining leave dates and planning working hours, as well as the employing institution.

Among Romanian participants, JSS mean scores differed significantly in relation to taking breaks from work, the ability to use legal leave at preferred times, and marital status disadvantages in selecting leave dates and planning working hours. GRAS mean scores varied according to gender-related disadvantages in choosing leave dates. MS mean scores showed significant differences associated with taking a break from work, the ability to use legal leave at preferred times, and marital status disadvantages in scheduling working hours.

As shown in Table 4, total JSS scores were correlated with total MS scores among participants from Türkiye, while no significant associations were identified between GRAS and MS or between JSS and GRAS. In contrast, among Romanian participants, significant correlations were observed between all three scales (JSS, GRAS, and MS).

A hierarchical multiple linear regression analysis was conducted to examine predictors of job satisfaction measured by the Job Satisfaction Scale (JSS) (Table 5).

In Model 1, mobbing (MS), gender role attitudes (GRAS), and country were included. The model was statistically significant and explained a substantial proportion of the variance in job satisfaction. MS and country emerged as significant predictors, whereas GRAS was not associated with JSS.

In Model 2, demographic and occupational variables (gender, age, years of professional experience, and profession) were added to control for potential confounding due to differences in occupational composition across countries. A statistically significant improvement in model fit and an increase in the proportion of explained variance were observed.

In the final model, MS, country, age, and years of professional experience remained significant predictors of JSS, whereas GRAS, gender, and profession were not. Specifically, higher levels of mobbing were associated with lower job satisfaction. Participants working in Romania reported higher job satisfaction compared to those in Türkiye. However, these findings should be interpreted cautiously, given differences in occupational composition across countries. Age showed a positive association with job satisfaction, whereas longer professional experience was negatively associated with it.

## 4. Discussion

### 4.1. Interpretation of the Main Findings

This cross-sectional study offers novel cross-cultural insights into workplace mobbing, gender-related perceptions, and job satisfaction among radiation workers in Türkiye and Romania. Consistent with previous research in healthcare settings, the findings indicate that workplace mobbing remains a prevalent psychosocial risk factor. However, the present study extends existing knowledge by focusing on a highly specialized occupational group and on both psychological and physical occupational stressors. Analysis of the scale scores of radiation workers from Türkiye and Romania shows that a range of socio-demographic and work-life factors influences job satisfaction, gender roles, and professional satisfaction.

### 4.2. Comparison with Other Studies

Gender roles are socially constructed from birth and shaped by cultural norms [31]. The literature shows that gender-based power inequalities are widely accepted in many societies, often contributing to the normalization of violence against women [32]. Although these power inequalities between genders make women feel inadequate, health workers have stated that the power inequality that men apply to women is socially structured and passed down from generation to generation [33]. In this context, the present study revealed cross-national differences in gender roles between Türkiye and Romania.

With respect to GRAS, findings from Türkiye indicate that gender role attitudes are significantly associated with gender and perceptions of gender-based disadvantage, suggesting a measurable impact on working life. Reported disadvantages during recruitment and in determining leave schedules point to limitations in workplace gender equality policies. The higher GRAS scores observed among Turkish participants may reflect more pronounced or clearly defined gender role expectations. These findings are consistent with previous research suggesting that women are often confined to traditional roles, such as motherhood, and are not fully recognized as individuals in society [34]. Gender role attitudes continue to associate women with domestic responsibilities and men with professional and managerial roles [35], contributing to workplace inequalities and occupational segregation [36]. A Romanian study on gendered perceptions of domestic life and quality of life [37], which used a questionnaire survey (1007 participants), found that some rural areas exhibit higher levels of gender bias than urban areas. The lower scores of Romanian participants may reflect less clear or more homogeneous perceptions of gender roles. These findings may reflect descriptive differences in work-life and organizational characteristics between the two country samples. Gender role perceptions are clearer, and relationships between co-workers are stronger in Türkiye. In Romania, interference with private life and lower job satisfaction are two important workplace issues.

Gender equality remains a key determinant of health, as gender roles influence behaviours and access to opportunities [33,38].

Regarding mobbing (MS), Romanian participants reported less exposure than those in Türkiye. Previous research has shown high levels of workplace mobbing and verbal violence among healthcare professionals, which negatively impact job satisfaction and retention [39,40]. In Romania, the lack of specific legislation on mobbing may affect how it is recognized and managed [41]. Here, mobbing was linked to the use of legal leave and disadvantages related to marital status, highlighting the importance of work–life balance. Organizational practices concerning working hours and leave may need improvement. Interestingly, Romanian participants reported higher job satisfaction, suggesting differences in working conditions or institutional support.

In Türkiye, mobbing was linked to gender, career pauses, and perceptions of disadvantage related to gender and marital status. Those who experienced career interruptions scored lower, highlighting the importance of employment stability. Challenges in accessing legal leave and organizing work hours suggest a need for more inclusive workplace policies, despite existing legal frameworks [1]. Lower job satisfaction may result from unmet expectations or poorer working conditions. Previous studies indicate that healthcare workers in Türkiye often report lower job satisfaction, underscoring the need for improved motivation and retention strategies [42,43].

For Turkish participants, job satisfaction was associated with how long they had been employed, their access to legal leave, and any perceived gender or marital status disadvantages, highlighting the importance of equitable working conditions. Dissatisfaction primarily stemmed from the work environment and income [44], supporting evidence that demographic and occupational factors affect job satisfaction [45].

Among Romanian participants, job satisfaction (JSS) was associated with career interruptions and disadvantages related to marital status, pointing to similar underlying factors. Those who experienced career breaks reported lower satisfaction, emphasizing the importance of stable work conditions. JSS scores among Romanians were significantly affected by perceptions of work disruptions and marital status, indicating that these factors influence job satisfaction [46,47]. The lower scores among those who took breaks suggest that continuous employment and stable working conditions are important for Romanian workers.

Different correlation patterns between GRAS, MS, and JSS were observed across the two country samples. In Türkiye, job satisfaction was more closely tied to workplace conditions, particularly mobbing and interpersonal relationships [48,49]. In contrast, correlations involving gender role attitudes were also observed in the Romanian sample.

Correlation analysis supports these results. In Türkiye, mobbing negatively correlates with job satisfaction, consistent with research linking workplace bullying to reduced motivation and increased emotional exhaustion [50]. In Romania, significant correlations were observed among the three variables, indicating potentially different contextual patterns compared with the Turkish sample [51].

Regression analysis revealed that mobbing is the most significant predictor of job satisfaction, with increased mobbing correlating with decreased satisfaction [52]. Moreover, variables such as country, age, and professional experience were important predictors. The higher satisfaction levels observed among Romanian participants could reflect organizational differences. Age showed a positive association with job satisfaction, whereas extensive professional experience was negatively associated, potentially due to accumulated stress [53]. Gender role attitudes did not significantly influence job satisfaction, suggesting that structural workplace factors have a more direct effect.

These findings may reflect descriptive differences in organizational and contextual characteristics between the two country samples. In the Turkish sample, job satisfaction was primarily correlated with workplace conditions, whereas in the Romanian sample, correlations involving gender role attitudes were also observed. Consequently, workplace interventions in both countries should prioritize improving workplace conditions and reducing mobbing, while also considering gender-related workplace perceptions within their organizational contexts. In specialized areas like medical imaging, radiotherapy, and nuclear medicine, psychosocial issues such as mobbing, job satisfaction, and gender inequality can indirectly affect professional performance. Although clinical outcomes weren’t measured, these findings highlight the importance of addressing psychosocial work environments across these fields.

### 4.3. Strengths and Limitations

This research has several strengths. It focuses on radiation workers, an occupational group that has been relatively underrepresented in research on workplace mobbing and psychosocial risks. The cross-cultural design, comparing participants from Türkiye and Romania, provides valuable comparative insights into how cultural and organizational contexts may influence workplace mobbing, gender-related perceptions, and job satisfaction. The structured questionnaire enabled standardized data collection across both countries, facilitating comparability and consistent responses. Including multiple psychosocial dimensions within a single analytical framework provides a more comprehensive understanding of workplace well-being in radiology settings.

Another important limitation of this study is the unequal occupational composition between the Türkiye and Romania samples. While technicians constituted the majority of the Turkish sample, medical physicists represented the largest professional group in the Romanian sample. Therefore, some differences interpreted as country-related may partially reflect differences in professional role, workplace responsibilities, educational background, organizational hierarchy, or job autonomy. Although profession was statistically controlled in the analyses, the findings should be interpreted cautiously. Future studies with more homogeneous occupational distributions and larger samples are recommended.

A limitation of this study is that the cross-sectional design precludes causal inference about the relationships among workplace mobbing, gender perception, and job satisfaction. The correlational nature of the data makes it difficult to establish causal relationships among the variables. Another limitation is that the data were self-reported, which may introduce reporting or social desirability bias. In addition, the use of convenience sampling and online self-report data collection may limit the representativeness and generalizability of the findings. Participants were recruited through professional online networks and voluntary participation, which may have introduced self-selection bias. Furthermore, the relatively small sample size may have reduced statistical power and limited the generalizability of the findings.

### 4.4. Practical Implications

The results highlight the need for institutional and national policies to address gender inequality and workplace harassment in medical imaging, radiotherapy and nuclear medicine departments. Greater transparency in task assignment, shift scheduling and leave planning can reduce perceptions of discrimination and improve job satisfaction. Clear reporting mechanisms and zero-tolerance policies for psychological harassment are important for employees, especially in units where stress levels are high. Interventions such as gender-sensitive leadership training, psychosocial risk monitoring and structured support programmes can reduce harassment-related stress and improve employee well-being. The differences between Türkiye and Romania indicate that policies should be tailored to specific organizational and socio-cultural contexts. Promoting workplace equity is both an ethical priority and a key factor in maintaining safe and effective services in hospital imaging, radiotherapy and nuclear medicine departments. The differences observed between Türkiye and Romania, particularly regarding mobbing exposure, job satisfaction, perceptions of gender-related disadvantage, and difficulties with leave planning and work organization, indicate that workplace policies and psychosocial interventions should be tailored to the specific organizational and socio-cultural contexts of each country. Promoting workplace equity is both an ethical priority and a key factor in maintaining safe and effective services in hospital imaging, radiotherapy and nuclear medicine departments.

## 5. Conclusions

This study suggests that workplace mobbing, gender role attitudes, and job satisfaction are interrelated among healthcare radiation workers in Türkiye and Romania. Mobbing emerged as the most consistent predictor of job satisfaction across the analyses, whereas associations involving gender role attitudes appeared to be more context-dependent and should be interpreted cautiously. Psychosocial risks extend beyond workload and may be associated with organizational and socio-cultural factors.

Healthcare institutions could adopt inclusive policies that ensure workplace transparency, provide gender awareness training, support women in leadership positions, and establish reliable systems for reporting harassment.

Addressing harassment and gender inequality is both an ethical obligation and a strategic priority for maintaining workforce well-being and safe, high-quality healthcare services.

## Figures and Tables

**Table 1:** Distribution of healthcare radiation workers descriptive characteristics.

Variables	Türkiye *n* (%)	Romania *n* (%)
**Gender**		
Female	66 (63.5%)	62 (71.3%)
Male	38 (36.5%)	25 (28.7%)
**Marital status**		
Married	49 (47.1%)	44 (50.6%)
Single	52 (50.0%)	35 (40.2%)
Divorced	3 (2.9%)	4 (4.6%)
Lives separately	0	4 (4.6%)
**Do you have children?**		
Yes	33 (31.7%)	40 (46%)
No	71 (68.3%)	47 (54%)
**Have you ever taken a break from your working life?**		
Yes	37 (35.6%)	27 (31%)
No	67 (64.4%)	60 (69%)
**Profession**		
Technician	99 (95.2%)	12 (13.8%)
Medical Physicist	2 (1.9%)	62 (71.3%)
Nurse	1 (1.0%)	5 (5.7%)
Medical Doctor	2 (1.9%)	8 (9.2%)
**Years of work experience?**		
1–5	39 (37.5%)	22 (25.3%)
6–10	16 (15.4%)	15 (17.2%)
≥11	49 (47.1%)	50 (57.5%)
**Have you experienced any disadvantages due to your gender during the recruitment process?**		
Yes	27 (26.0%)	13 (14.9%)
No	77 (74.0%)	74 (85.1%)
**Can you use your legal leaves (annual, current, etc.) on any date you want?**		
Yes	51 (49.0%)	73 (83.9%)
No	53 (51.0%)	14 (16.1%)
**Have you experienced any disadvantages related to your gender when determining your leave dates?**		
Yes	17 (16.3%)	8 (9.2%)
No	87 (83.7%)	79 (90.8)
**Have you experienced any disadvantages regarding your marital status when determining your leave dates?**		
Yes	29 (27.9%)	8 (9.2%)
No	75 (72.1%)	79 (90.8)
**Have you experienced any disadvantages related to your gender when planning your working hours?**		
Yes	21 (20.2%)	7 (8.0%)
No	83 (79.8%)	80 (92.0%)
**Have you experienced any disadvantages regarding your marital status when planning your working hours?**		
Yes	33 (31.7%)	9 (10.3%)
No	71 (68.3%)	78 (89.7%)
**The institution you currently work for**		
Public	33 (31.7%)	31 (35.6%)
University	18 (17.3%)	16 (18.4%)
Private	53 (51.0%)	40 (46.0%)
**Age**	**Mean ± SD**	**Mean ± SD**
	29.77 ± 9.13	35.73 ± 10.80

Bold formatting is used only for the main categorical variables (section headings) to improve the readability and organization of the table.

**Table 2:** Total and Subdimension Mean Scores of the GRAS, MS, and JSS of the healthcare radiation workers.

Scale and SubDimension	Türkiye (*n* = 104)			Romania (*n* = 87)		
	Mean ± SD	Values from the Scales		Mean ± SD	Values from the Scales	
		Min	Max		Min	Max
† GRAS_total	163.10 ± 15.66	109	186	151.42 ± 17.33	99	178
Female gender roles	33.13 ± 4.80	18	40	29.31 ± 3.59	22	38
Egalitarian gender roles	37.32 ± 3.24	20	40	32.93 ± 5.34	16	40
Marriage gender roles	37.28 ± 3.28	23	40	35.25 ± 4.65	21	40
Traditional gender roles	28.97 ± 5.31	11	36	28.14 ± 3.76	18	36
Male gender roles	26.12 ± 3.33	17	30	25.56 ± 3.59	14	30
⁂ MS_total	77.41 ± 26.59	38	138	72.03 ± 23.49	38	146
Relationships with co-workers	37.13 ± 14.95	17	73	32.45 ± 12.06	17	65
Threats and harassment	12.68 ± 4.43	7	29	11.94 ± 4.30	7	28
Interference with private life	6.66 ± 2.51	4	15	17.19 ± 6.13	8	33
Commitment to work	3.99 ± 1.80	2	9	6.67 ± 2.17	4	16
Obstacles related to work and career	16.94 ± 6.86	8	36	3.75 ± 1.43	2	8
‡ JSS_total	15.65 ± 5.44	5	25	19.21 ± 2.89	12	25

⁂ MS: Mobbing Scale, ‡ JSS: Job Satisfaction Scale, † GRAS: Gender Role Attitude Scale.

**Table 3:** (**a**). Comparison of JSS, GRAS, and MS scores by demographic characteristics. (**b**). Comparison of JSS, GRAS, and MS Scores by workplace conditions and gender-related experiences.

(a)						
Variables	JSS		GRAS		MS	
	Türkiye	Romania	Türkiye	Romania	Türkiye	Romania
	Mean ± SD	Mean ± SD	Mean ± SD	Mean ± SD	Mean ± SD	Mean ± SD
**Gender**						
Female	15.30 ± 5.30	19.11 ± 2.75	169.33 ± 10.74	154.59 ± 14.55	81.39 ± 27.32	73.83 ± 21.98
Male	16.26 ± 5.69	19.48 ± 3.28	152.28 ± 17.05	143.56 ± 21.15	70.50 ± 24.10	67.56 ± 26.83
**Test score**	t = −0.865 *p* = 0.389	t = −0.532 *p* = 0.596	t = 5.558 *p* = 0.000 *	t = 2.390 *p* = 0.023 *	t = 2.042 *p* = 0.044 *	t = 1.130 *p* = 0.262
**Effect size**			**d = 1.24**	**d = 0.57**	**d = 0.47**	
**Have you ever taken a break from your working life?**						
Yes	15.16 ± 5.02	18.29 ± 3.17	162.56 ± 14.11	149.00 ± 20.02	85.24 ± 26.29	81.18 ± 25.20
No	15.92 ± 5.67	19.63 ± 2.69	163.40 ± 16.55	152.51 ± 16.04	73.08 ± 25.95	67.91 ± 21.66
**Test score**	t = −0.683 *p* = 0.496	t = −2.026 *p* = 0.046 *	t = −0.259 *p* = 0.796	t = −0.874 *p* = 0.384	t = 2.276 *p* = 0.025 *	t = 2.511 *p* = 0.014 *
**Effect size**		**d = 0.49**			**d = 0.47**	**d = 0.58**
**Profession**						
Technician	15.63 ± 5.52	20.08 ± 2.71	162.86 ± 15.95	143.58 ± 21.42	77.09 ± 26.86	61.75 ± 7.00
Medical Physicist	16.00 ± 1.41	18.83 ± 2.90	172.00 ± 11.31	152.24 ± 15.37	95.50 ± 10.60	74.38 ± 2.89
Nurse	-	18.60 ± 1.67	-	136.20 ± 23.77	-	77.00 ± 6.09
Medical Doctor	18.50 ± 3.53	21.25 ± 3.01	163.50 ± 4.94	166.37 ± 7.50	62.50 ± 16.26	66.12 ± 10.74
**Test score**	K = 0.888 *p* = 0.641	F = 2.182 *p* = 0.096	K = 0.759 *p* = 0.684	F = 4.661 *p* = 0.005 *	K = 2.647 *p* = 0.266	F = 1.226 *p* = 0.305
**Effect size**				**η^2^ = 0.14**		
**How many years have you been in years of work experience?**						
1–5	17.35 ± 5.44	18.36 ± 2.93	162.00 ± 17.25	151.72 ± 19.85	76.53 ± 28.71	76.09 ± 27.44
6–10	14.56 ± 5.27	18.80 ± 3.12	167.62 ± 10.63	146.46 ± 19.80	84.62 ± 27.17	75.53 ± 24.00
≥11	14.65 ± 5.25	19.72 ± 2.76	162.51 ± 15.72	152.78 ± 15.39	75.75 ± 24.77	69.20 ± 21.49
**Test score**	F = 3.196 *p* = 0.045 *	F = 1.900 *p* = 0.156	F = 0.795 *p* = 0.454	F = 0.765 *p* = 0.468	F = 0.700 *p* = 0.499	F = 0.855 *p* = 0.429
**Effect size**	**η^2^ = 0.06**					
(**b**)						
**Variables**	**JSS**		**GRAS**		**MS**	
	**Türkiye**	**Romania**	**Türkiye**	**Romania**	**Türkiye**	**Romania**
	**Mean ± SD**	**Mean ± SD**	**Mean ± SD**	**Mean ± SD**	**Mean ± SD**	**Mean ± SD**
**Have you experienced any disadvantages due to your gender during the recruitment process?**						
Yes	14.25 ± 4.83	17.92 ± 2.78	170.85 ± 10.45	146.4615 ± 22.22	87.14 ± 27.40	78.2308 ± 26.60
No	16.14 ± 5.58	19.4459 ± 2.87	160.38 ± 16.32	152.2973 ± 16.36	74.00 ± 25.61	70.9459 ± 22.93
**Test score**	t = −1.558 *p* = 0.122	t = 0.873 *p* = 0.081	t = 3.109 *p* = 0.002 *	t = −1.121 *p* = 0.265	t = 2.254 *p* = 0.026 *	t = 1.031 *p* = 0.305
**Effect size**			**d = 0.70**		**d = 0.51**	
**Can you use your legal leaves (annual, current, etc.) on any date you want?**						
Yes	17.78 ± 4.51	19.5479 ± 2.81	160.60 ± 15.90	152.6301 ± 16.74	66.74 ± 23.23	68.8767 ± 21.25
No	13.60 ± 5.50	17.5000 ± 2.79	165.50 ± 15.19	145.1429 ± 19.60	87.67 ± 25.74	88.5000 ± 28.34
**Test score**	t = 4.225 *p* < 0.001 *	t = 2.493 *p* = 0.015 *	t = −1.607 *p* = 0.111	t = 1.491 *p* = 0.140	t = −4.347 *p* < 0.001 *	t = −2.461 *p* = 0.026 *
**Effect size**	**d = 0.84**	**d = 0.73**			**d = 0.86**	**d = 0.78**
**Have you experienced any disadvantages related to your gender when determining your leave dates?**						
Yes	13.29 ± 5.83	18.3750 ± 2.77	169.35 ± 10.13	135.1250 ± 21.24	101.88 ± 26.21	71.6250 ± 31.99
No	16.11 ± 5.27	19.3038 ± 2.91	161.88 ± 16.29	153.0759 ± 16.14	72.63 ± 24.03	72.0759 ± 22.73
**Test score**	t = −1.983 *p* = 0.050	t = −0.862 *p* = 0.391	t = 2.477 *p* = 0.018 *	t = −2.910 *p* = 0.005 *	t = 4.522 *p* < 0.001 *	t = −0.051 *p* = 0.959
**Effect size**			**d = 0.53**	**d = 1.00**	**d = 1.15**	
**Have you experienced any disadvantages regarding your marital status when determining your leave dates?**						
Yes	12.62 ± 5.31	16.6250 ± 2.13	166.00 ± 13.59	149.8750 ± 21.40	95.06 ± 24.76	80.5000 ± 19.66
No	16.82 ± 5.05	19.4810 ± 2.84	161.98 ± 16.34	151.5823 ± 17.02	70.58 ± 24.15	71.1772 ± 23.79
**Test score**	t = −3.753 *p* < 0.001 *	t = −2.755 *p* = 0.007 *	t = 1.174 *p* = 0.243	t = −0.264 *p* = 0.792	t = 4.604 *p* < 0.001 *	t = 1.070 *p* = 0.288
**Effect size**	**d = 0.81**	**d = 1.03**			**d = 1.00**	
**Have you experienced any disadvantages related to your gender when planning your working hours?**						
Yes	13.47 ± 6.19	17.14 ± 3.23	163.28 ± 15.71	139.57 ± 20.79	95.76 ± 28.63	84.00 ± 31.49
No	16.20 ± 5.12	19.40 ± 2.81	163.06 ± 15.74	152.46 ± 16.75	72.77 ± 24.09	70.98 ± 22.62
**Test score**	t = −2.086 *p* = 0.039 *	Z = −1.609 *p* = 0.108	t = 0.059 *p* = 0.953	Z = −1.787 *p* = 0.074	t = 3.757 *p* < 0.001 *	Z = −1.335 *p* = 0.182
**Effect size**	**d = 0.50**				**d = 0.89**	
**Have you experienced any disadvantages regarding your marital status when planning your working hours?**						
Yes	12.78 ± 4.98	17.11 ± 3.01	163.39 ± 12.01	151.11 ± 23.60	93.66 ± 24.30	91.44 ± 22.23
No	16.98 ± 5.15	19.46 ± 2.80	162.97 ± 17.17	151.46 ± 16.66	69.85 ± 24.25	69.79 ± 22.71
**Test score**	t = −3.90 *p* < 0.001 *	Z = −2.145 *p* = 0.032 *	t = 0.145 *p* = 0.885	Z = −0.272 *p* = 0.786	t = 4.655 *p* < 0.001 *	Z = −2.643 *p* = 0.008 *
**Effect size**	**d = 0.82**	**r = 0.23**			**d = 0.98**	**r = 0.28**
**The institution you currently work for.**						
Public	16.27 ± 4.89	18.90 ± 2.94	160.66 ± 17.68	150.29 ± 16.75	66.57 ± 23.63	73.22 ± 24.63
University	16.38 ± 5.86	19.12 ± 2.33	157.38 ± 18.55	152.93 ± 14.64	78.05 ± 30.02	82.93 ± 23.17
Private	15.01 ± 5.61	19.50 ± 3.09	166.56 ± 12.40	151.70 ± 19.02	83.94 ± 25.39	66.75 ± 21.57
**Test score**	F = 0.735 *p* = 0.482	K = 0.561 *p* = 0.755	K = 3.004 *p* = 0.054	K = 0.470 *p* = 0.791	F = 4.650 *p* = 0.012 *	K = 5.949 *p* = 0.051
**Effect size**					**η^2^ = 0.08**	

* *p* < 0.05 t: independent *t*-test F: One-way ANOVA test Z: Mann-Whitney U test K: Kruskal-Wallis test d: Cohen’s d effect size. Effect sizes were reported only for statistically significant group comparisons. Cohen’s d was reported for independent-samples *t*-tests, η^2^ for ANOVA, and r for Mann–Whitney U tests.

**Table 4:** Pearson’s Correlation between JSS, GRAS, and MS in radiation healthcare workers.

Scales	Türkiye				Romania		
	JSS		GRAS	MS	JSS	GRAS	MS
**‡ JSS**	r	1			1		
**† GRAS**	r	0.023	1		0.346 * [0.14, 0.52]	1	
**⁂ MS**	r	−0.551 * [−0.67, −0.40]	−0.006	1	−0.501 * [−0.64, −0.33]	−0.398 * [−0.56, −0.20]	1

* *p* < 0.001 ⁂ MS: Mobbing Scale, ‡ JSS: Job Satisfaction Scale, † GRAS: Gender Role Attitude Scale. Values in brackets represent 95% confidence intervals.

**Table 5:** Hierarchical Multiple Linear Regression Predicting Job Satisfaction (JSS).

Variables	Model 1 β	*p*	Model 2 β	95% CI (B)	*p*
MS	−0.484	<0.001	−0.492	−0.116, −0.071	<0.001
GRAS	0.028	0.653	0.021	−0.048, 0.066	0.762
Country (Romania vs. Türkiye)	0.329	<0.001	0.264	0.974, 4.103	0.002
Gender	–	–	−0.007	−1.508, 1.349	0.921
Age	–	–	0.170	0.165, 2.047	0.021
Years of Professional Experience	–	–	−0.215	−1.893, −0.413	0.002
Profession	–	–	0.062	−0.946, 2.279	0.412
Model Fit		Model 1		Model 2	
R^2^		0.374		0.409	
Adjusted R^2^		0.364		0.387	
ΔR^2^		–		0.035	
F		37.30 ***		18.54 ***	

JSS = Job Satisfaction Scale. MS = Mobbing Scale. GRAS = Gender Roles Attitude Scale. Country coded as 1 = Türkiye, 2 = Romania. β = standardized regression coefficient. *** *p* < 0.001. Reference categories were Türkiye for country, female for gender, and technician for profession. Dummy-coded categorical variables were compared with the stated reference categories.

## Data Availability

The data presented in this study are available on request from the corresponding author due to the sensitive topic.

## Abbreviations

The following abbreviations are used in this manuscript:

GRAS	Gender Roles Attitude Scale
JSS	Job Satisfaction Scale
MS	Mobbing Scale
